# Effectiveness and cost-effectiveness of body psychotherapy in the treatment of negative symptoms of schizophrenia – a multi-centre randomised controlled trial

**DOI:** 10.1186/1471-244X-13-26

**Published:** 2013-01-14

**Authors:** Stefan Priebe, Mark Savill, Ulrich Reininghaus, Til Wykes, Richard Bentall, Christoph Lauber, Paul McCrone, Frank Röhricht, Sandra Eldridge

**Affiliations:** 1 Unit for Social and Community Psychiatry, Queen Mary University of London, Newham Centre for Mental Health, London, E13 8SP, UK; 2 Institute of Psychiatry, Kings College London, De Crespigny Park, London, SE5 8AF, UK; 3 Department of Psychiatry, University of Liverpool, Waterhouse Building, 1-5 Brownlow Street, Liverpool, L69 3GL, UK; 4 King's College London, Health Service and Population Research Department, Institute of Psychiatry, De Crespigny Park, London, SE, UK; 5 Centre for Health Sciences, Barts and the London School of Medicine and Dentistry, Queen Mary University of London, Abernethy Building, 2 Newark Street, London, E1 2AT, UK

## Abstract

**Background:**

Negative symptoms of schizophrenia are frequently associated with poor long term outcomes. Established interventions have little, if any, positive effects on negative symptoms. Arts Therapies such as Body Psychotherapy (BPT) have been suggested to reduce negative symptoms, but the existing evidence is limited. In a small exploratory trial a manualised form of group BPT led to significantly lower negative symptom levels both at the end of treatment and at 4 months follow-up as compared to supportive counseling. We designed a large multi-site trial to assess the effectiveness of a manualised BPT intervention in reducing negative symptoms, compared to an active control.

**Methods/Design:**

In a randomised controlled trial, 256 schizophrenic outpatients with negative symptoms will be randomly allocated either to BPT or Pilates groups. In both conditions, patients will be offered two 90 minutes sessions per week in groups of about 8 patients over a period of 10 weeks. Outcomes are assessed at the end of treatment and at six months follow-up. The primary outcome is severity of negative symptoms, as measured by the Positive and Negative Symptom Scale (PANSS), whilst a range of secondary outcome measures include general psychopathology, social contacts, and quality of life. We will also assess the cost-effectiveness of the intervention.

**Discussion:**

The study aims to evaluate the effectiveness of a promising form of group therapy which may help alleviate negative symptoms that are associated with unfavourable long-term outcomes and have so far been difficult to treat. If the trial is successful, it will add a new and effective option in the treatment of negative symptoms. Group BPT is manualised, might be attractive to many patients because of its unusual approach, and could potentially be rolled out to services at relatively little additional cost.

**Trial registration:**

Current Controlled Trials ISRCTN84216587

## Background

Despite improvements of anti-psychotic medication and psychological treatments, schizophrenia patients often experience persistent symptoms and full remissions are infrequent. Patients often do not adhere to existing pharmacological and psychological treatments and even when they do, currently available treatments have an only limited effect. This applies in particular to negative symptoms which are frequently associated with poor long term outcomes.

The National Institute for Clinical Excellence (NICE) reviewed the literature on the treatment of negative symptoms and identified six trials using different forms of ‘arts therapies’. Arts therapies is an umbrella term including therapies that have central non-verbal components, such as Body Psychotherapy (BPT). NICE suggested that arts therapies are effective in reducing negative symptoms as compared to any other control. The Guideline Development Group recommended that “further large-scale investigations of arts therapies should be undertaken to increase the current evidence base” [1] (p199). In particular, “an adequately powered trial should be conducted to investigate the clinical and cost-effectiveness of arts therapies as compared to an active control in people with schizophrenia” [1] (p200).

BPT refers back to a long tradition in psychiatry. A first trial in schizophrenia patients was published in 1965 showing a significant improvement of the treatment group, as compared to controls in affective contact, motility and general functioning [2]. Four further controlled studies - three of them randomised - compared forms of BPT with non-specific attention, music therapy or fitness training. The results suggest favourable effects of the experimental treatments on a range of outcome variables, including some indicators of negative symptoms [2-5]. However, all these studies, which were exclusively conducted before 1980, have serious methodological shortcomings such as small sample sizes, vaguely defined outcome criteria, no systematic assessment of psychopathology, no recording of medication, and no intention-to-treat analysis.

A more recent exploratory randomised controlled trial tested manualised BPT in out-patients with persistent negative symptoms of schizophrenia [6]. Compared to a control group receiving supportive counselling, patients in the experimental group showed a significant improvement of negative symptoms. The effect size was large, and maintained at a 4 month follow up. The major limitations of that study were that it was a small exploratory trial based at one site and with only one BPT therapist, and that the control condition was supportive counselling, which turned out to be unattractive to patients and meant that the non-specific effects of physical activity could not be controlled for. In addition, the exploratory study did not provide sufficient evidence for the cost-effectiveness of BPT. Following these findings, an open uncontrolled trial of manualised BPT was conducted by the same authors with different therapists which yielded similar results [7], however to date there have been no other rigorous trials on BPT or a similar method in the treatment of schizophrenia [8].

The specific components of the BPT under investigation are a) the focus on body experience at a cognitive and emotional level b) the facilitation of emotional group interactions, and c) the link between movement and emotion. The manual for BPT [9], which was applied in the exploratory trial and will also be used in this pragmatic trial, intends to utilise the three specific mechanisms. Common components utilised in both interventions will include the non-specific effects of non-emotional group interactions, therapist/instructor attention, and physical activity.

In a review into physical exercise for schizophrenia [10], physical activity groups were found to significantly improve depression scores and negative symptoms in comparison to treatment as usual, but in a study which used physical activities as an active control designed to evaluate the effectiveness of yoga therapy [11] yoga was found to result in a significantly greater reduction in negative symptoms in comparison to physical activity alone. This study suggests that physical activity groups can be used as an active control, fulfilling the requirement laid down by NICE for a ‘sham condition’, but with physical activity groups being found to have a significant effect on negative symptoms this study may under-report any effect of the intervention compared to the usual treatment patients are likely to receive.

Beyond the expected clinical effectiveness, there are three characteristics of BPT that make it particularly beneficial for service providers: 1) as a group based method, BPT is relatively inexpensive; 2) BPT can be flexibly combined with other treatment methods including all pharmacological interventions; 3) because BPT is so distinct from conventional treatment methods, it can appeal to patients who are difficult to engage in other forms of treatment. The latter point is underlined by findings of a qualitative evaluation of the exploratory trial [7] in which patients fed back most positively about both its focus and methods.

## Methods

### Study Design

In a randomised controlled trial, schizophrenia patients with negative symptoms will be randomly allocated to groups for BPT or Pilates as an active control. Both conditions are in addition to treatment as usual and will be delivered in groups of about 8 patients each. There will be 3 data collection points; before treatment, after the 10 week treatment period, and after a 6 month follow-up period. Each therapist in either condition will run a maximum of two groups, and in each group they will be assisted by a co-facilitator. The trial cannot be fully “blind” because clinicians and patients cannot be masked towards the allocation of the patients to the experimental or control group. However, researchers who conduct the outcome assessments will be masked to the allocation of patients, and the eligibility and baseline assessments will occur pre-randomisation. All the analysis will be conducted blind to the participant allocation.

Originally, the plan was to randomise 16 patients at a time in each site, eight to intervention and eight to control, but this was changed for logistic reasons. We will now recruit a larger number, i.e. up to 20 patients in one batch, to ensure we have a sufficient number of participants randomised to cover any dropout between the eligibility and baseline assessment phases. After the baseline assessments, participants will be randomly allocated to the intervention or control group in batches using randomly permuted blocks of 4 and 6, starting each batch at the start of a new block to preserve balance. Conducting the randomisation in this manner allows us to randomise all the participants in one batch either at the same time or over two time points, allowing the research team more time to finalise the logistics involved with getting all the participants to the groups at the correct times.

BPT and Pilates groups will both run for 10 weeks. After the 10 week intervention period and after a further 6 month follow-up patients will be interviewed by the researchers and outcomes will be assessed. These assessments will be conducted either on NHS sites or in the community, ideally in the same location as the original assessments.

### Planned interventions

Patients in the experimental group will receive 20 sessions of BPT which will be delivered in groups of approximately 8 patients over a 10 week period. Each session will take 90 minutes.

BPT is manualised with the following components: 1) overcoming communication barriers through non-verbal techniques; 2) re-focussing cognitive and emotional awareness towards the body; 3) stimulating activity and emotional responsiveness; 4) exploring physical potentials; 5) focussing on strength and experiencing the body as a source of creativity, reliability, pleasure and self-expression; 6) modifying dysfunctional self-perception and addressing body-related psychopathological features such as boundary loss, somatic depersonalisation, and body schema disturbances.

Each BPT session contains five discrete sections; 1) the opening circle, which is used to describe feelings and energy levels; 2) a warm-up section, where participants stand in a circle and warm up using different body parts and movements; 3) a structured task section, which includes exercises such as mirroring each other’s movements and creating body image sculpture in partners; 4) a creative movements section, which includes exercises such as creating group sculptures and reflecting on perceptions and emotions; 5) a closing circle, used to reflect on the group experience and to refocus on the self with body oriented exercises. More detail on this is available in the manual [9].

All therapists will be accredited dance-movement psychotherapists with additional training in applying the manual of body psychotherapy for patients with negative symptoms of schizophrenia, as it was used in the exploratory trial [6]. Regular supervision will be provided 3 times within each 10 week treatment period. Adherence to the manual will be assessed by the author of the manual based on videotapes of the sessions.

The active control condition of Pilates groups will be delivered with the same frequency and length of sessions and overall duration as BPT, i.e. there will be two sessions per week over a ten week period and each session will last up to 90 minutes. Pilates groups will be described to patients as a fitness and physical health intervention so that in the recruitment process patients consent to participate in one of two interventions that share several elements, most importantly the format and physical activities. This is intended to limit the risk of different acceptance rates of the two interventions once patients are informed about their allocation.

The venues for the Pilates groups will be similar rooms as those used for BPT. Pilates groups will be delivered by qualified instructors supported by a co-facilitator, and follow the established guidelines for such groups. A brief manual for the Pilates groups was written by qualified instructors involved in the trial, in collaboration research team, using the Pilates Union Matwork Manual as a guide [12]. The Trial manual contains instructions on how to run the groups, as well as references to material describing the exercises in detail. Group interactions will occur as they commonly do when patients are together, but the instructor will not actively encourage or structure them. Instructors will pay attention to patients and respond to them without addressing or verbalising emotions. As an active control condition in this trial, Pilates will therefore provide moderate physical activity, instructor attention and non-emotional group interaction, but not the specific components of BPT which are the focus on body experience, the facilitation of emotional group interactions, and linking movement and emotional experience. In both conditions the same therapist or instructor will be in charge of a maximum of two groups.

### Inclusion criteria

The inclusion criteria for community patients receiving mental health care in services in the National Health Service (NHS) are as follows: 

Being aged between 18 and 65 years.

Having an established diagnoses of schizophrenia according to DSM-IV.

Having had symptoms of schizophrenia for at least 6 months.

Having Scores of ≥18 on the negative symptoms subscale of the Positive and Negative Symptom Scale; the PANSS [13].

Not changing the type of anti-psychotic medication prescribed for at least 6 weeks prior to recruitment (although dosage may change).

A willingness to participate in groups for BPT or physical activity.

An ability to give written informed consent.

### Exclusion criteria

The exclusion criteria are: 

Severe physical disability preventing patients from participating in groups for BPT or Pilates.

Insufficient command of English so that outcomes cannot be reasonably assessed in English.

A physical condition that makes participation in either BPT or Pilates impossible or potentially. harmful.

### Outcome measures

The primary outcome criterion for the effectiveness of BPT is the level of negative symptoms as assessed on the PANSS [13].

The secondary outcomes are: 

The levels of general psychopathology and positive symptoms, measured by the PANSS [13].

Subjective quality of life, measured by the Manchester Short Assessment of Quality of Life [14].

A measure of the amount activities the participant has engaged in during the previous week, using items from the Time Use Survey [15].

The participants level of treatment satisfaction in the intervention, measured by the Client Satisfaction Questionnaire [16].

A measure of the participants objective social situation, recorded by the SIX [17].

Extrapyramidal symptoms, using the Simpson-Angus Scale [18].

A secondary measure of negative symptoms which has been developed recently and attempts to differentiate between measures of emotional experience and emotional expression (Clinical Assessment interview for Negative Symptoms; the CAINS, [19]).

A measure of nonverbal communication/gesture behaviour, based on videotapes of the assessments using the NEUROGES-ELAN system [20].

A measure of Depression (the Calgary Depression Scale [21]).

The level of social contacts the participant has made in the past month, measured through using sections of the Social Network Scale [22].

A measure of cost-effectiveness using the Client Service Receipt Inventory (CSRI, [23]).

A measure of Quality of life adjusted years, based on the EQ-5D [24].

All criteria will be obtained at pre-treatment baseline, at the end of treatment, and after a 6 month follow-up. Our primary hypothesis relates to endpoints at the end of treatment.

The number of therapy sessions attended by patients in both groups will be recorded. Costs of BPT and Pilates will be calculated taking into account the staff time involved in delivering therapy, training received and supervision (including time of both therapists and those providing training/supervision). The training costs will be apportioned over the period of time in the trial during which therapy is delivered. However, the benefits of training are likely to run beyond the trial and therefore sensitivity analyses will involve apportioning training costs over longer periods. Other service use information (including health and social care inputs, medication and informal care) will be collected for the 6 months prior to treatment, the treatment period of 10 weeks, and the 6 month follow-up period using the CSRI [23]. Service use data will be combined with nationally applicable unit costs. Informal care costs will be based on the unit cost of a homecare worker, with wage rates for employed carers used as an alternative in sensitivity analyses. Costs will be reported in line with NICE guidance.

### Ethics Approval

The study has been approved by the Camden & Islington National Research Ethics Committee (REC reference 10/H0722/44)**.** All data will be stored in line with the Data Protection Act. All video-recorded data will be encrypted and password protected.

### Trial Steering Committee and Data Monitoring and Ethics Committee

Both a Trial Steering Committee (TSC) and Data Monitoring and Ethics Committee (DMEC) have been established.

### Recruiting participants and obtaining informed consent

In order to recruit participants clinicians will be asked to screen their caseloads to identify those eligible and appropriate for the study, and then to ask them for their consent to be approached by a research assistant. If the patient agrees, they will be contacted by a member of the research team, fully informed about the study, and asked for written informed consent. Once the participant has consented, the researcher will then conduct the eligibility assessment. Consent to take part in the trial can be withdrawn at any time, without any negative consequences, other than that patients would not have the potential benefits of further participation in the BPT or Pilates groups. As part of this process we will request consent for the pre and post intervention PANSS & CAINS interviews to be video recorded to enable independent researcher ratings as well as further analysis of the data. This is optional for patients, and if they do not wish to be video-recorded this will not impact on their participation in the study.

### Statistical analysis

The primary analysis will be an available case analysis following intention to treat principles. We will use mixed effect models as appropriate to the outcome, with fixed effects for the intervention, baseline level of outcome and centre, and random effects for therapy groups. For continuous outcomes we will use Stata’s *xtmixed* command, *xtmepoisson* for count outcomes, and *gllamm* for ordinal outcomes. We will assess whether there is a clustering effect due to therapist or instructor (some therapists or instructors will lead two groups), but will not take this element of clustering into account in the analysis.

We will use sensitivity analyses to explore the impact of any missing data, including extreme scenario sensitivity analyses, for example that those who are lost to follow up would have no improvement as result of any intervention. If there are sufficient missing values to warrant its use, we will use multiple imputation following an exploration of patterns of missingness. We expect to use the MAR (missing at random) assumption in this imputation. In case group dropout (as opposed to study dropout) is higher in one condition compared to the other, we will conduct a simple complier-average causal analysis with compliers defined by the active intervention. Compliance will be defined as attendance of five or more sessions of BPT.

### Economic evaluation

Cost data comparisons between the groups will be made using a bootstrap regression model (using 10,000 repetitions). Either bias-corrected or percentile 95% confidence intervals will be reported depending on the level of bias in the data. Cost-effectiveness and cost-utility will be established by combining the cost data (NHS/PSS and then all services) with change scores on the PANSS and QALYs respectively. In addition, cost-effectiveness acceptability curves (CEACs) will show the probability that BPT or Pilates is cost-effective for different values placed on unit-changes in outcome. The net benefit approach will be used (i.e. the monetary value of outcome change minus the service cost) and net benefit will then be used in bootstrapped regression models. The proportion of coefficients for each resample that are above zero will indicate the probability that BPT is the most cost-effective option. The values used for the CEAC based on QALY gains will be £0 to £100,000 in £5,000 increments, which includes the threshold of £20,000 to £30,000 used by NICE. We will also report values for PANSS improvements that are linked to BPT/Pilates having a 50%, 75% and 95% likelihood of being cost-effective.

Because this project uses an active control we are essentially looking at the cost-effectiveness of receiving BPT rather than Pilates. Both BPT and Pilates are delivered by a therapist/instructor and in groups so we will be costing both interventions. However, in routine practice BPT is likely to be considered as an additional service rather than as an alternative to other active therapies. In sensitivity analyses we will therefore apply lower unit costs (25%, 50%, 75% and 100% lower) for the active control in an attempt to replicate routine practice where no or limited physical activities are provided. In case BPT could be delivered by other professionals who are more or less expensive, we will also increase/decrease the unit cost of BPT by 25% and 50% in sensitivity analyses.

### Proposed sample size

**Figure 1:**
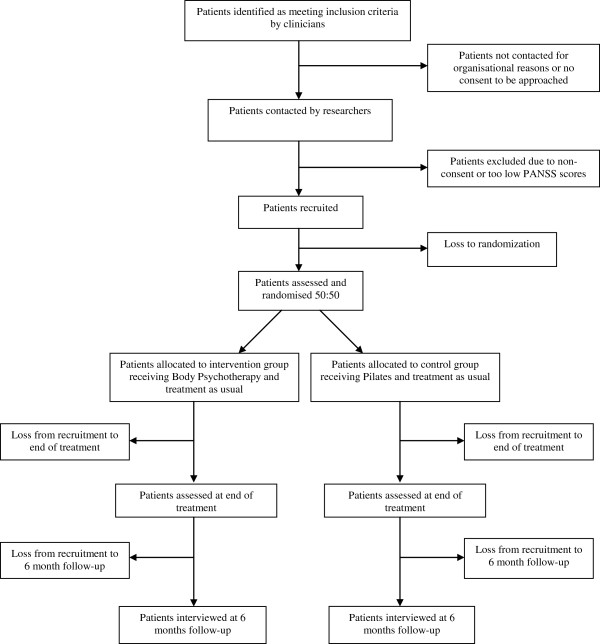
Flow Diagram.

A 20% reduction in the PANSS negative scale has been defined as clinically significant [25], which we would anticipate to be a difference of approximately 3 points given the eligibility criteria. To detect a difference of 3 points, with a standard deviation of 5, at alpha = 5%, and a power of 90%, 58 patients are required in each arm. Assuming an intra-cluster correlation coefficient for treatment group of 0.1, and 7 patients per group with analysable data at the end of treatment gives an inflation factor of 1.6 [24] leading to a required sample size of 93 in each arm. At six months we expect a loss to follow-up of 31%, resulting in 5.5 individuals per group on average and an inflation factor of 1.45; recruiting 128 per arm (16 groups of 8 in each arm) leaves 88 per arm at 6 months and 91% power to detect a difference of 3 points at this time point (see Figure 1). One hundred and twenty eight patients per arm, i.e. 16 groups of approximately 8 patients in each arm will give 94% power for the end of treatment analysis. We do not expect to recruit and allocate exactly eight individuals within each group but the variation in group size will be small and will not materially affect the power of the trial.

The estimated loss takes into account drop outs at three different phases of the study, i.e. a) between first interview and beginning of treatment, b) during treatment, and c) during the six months follow up period. Some patients will have to wait up to 5.5 months between giving consent and beginning of treatment. Within that period they could drop out because they change their mind about participation or experience a reduction of negative symptoms so they no longer meet the inclusion criteria. Based on the exploratory trial and clinical experiences in East London, we expect this dropout rate to be less than 10%. During the treatment phase we will have drop outs from treatment in both groups. In line with the intention to treat analysis we will try and follow up all patients who dropped out of treatment in either arm.

In the exploratory trial, the dropout rate from randomisation to 4 month follow-up was 35%. To achieve a lower dropout rate despite a longer follow-up period as compared to the exploratory trial, i.e. 6 instead of 4 month, we will take the following steps:

a) patients will be reimbursed for their expenses and time for each research interview;

b) at each interview (other than the final one at the end of the follow up period), research assistants will already arrange the meeting for the next interview and two weeks before the meeting send a reminder to the patient;

c) the same research assistant will conduct all four interviews with a patient so that a positive relationship can be established;

d) research assistants will work full time on the study so that they are flexible in accommodating the patients’ wishes for meetings at specific times and at specific places including the patients’ homes.

## Discussion

At present there is some limited evidence to suggest that BPT can help reduce the negative symptoms of schizophrenia. This is significant given these particular symptoms are associated with poorer outcomes, and are resistant to most other forms of treatment. The aim of this study is to build on the current evidence base and examine the effectiveness of BPT in a large multi-site trial, examining both the effectiveness and cost-effectiveness in comparison to an active control.

In comparison to the exploratory study which found a strong effect of BPT on negative symptoms [6], the most significant revision, other than the scale of the project, has been to replace the previous control condition of counselling with a structured Pilates group. This modification will allow us to test whether it is the specific components of BPT, i.e. the focus on body experience, the facilitation of emotional group interactions, and the link between movement and emotion, as opposed to the physical activity and the group format which reduces negative symptoms. One possible limitation of using Pilates as a control however is that the non-specific effects of a regular, structured group-intervention, with group facilitator attention and supported attendance as provided in Pilates groups is far from clear.

Implementing the trial design has a number of challenges. By definition, patients experiencing severe negative symptoms have high levels of social withdrawal and low motivation to engage in activities, meaning it might be very difficult to engage participants in study. In addition, the validity of assessing negative symptoms with established measures such as the PANSS has been questioned [26], whilst newer scales developed to address these concerns such as the CAINS [19] have not yet been extensively used.

If the trial does demonstrate the effectiveness and cost-effectiveness of BPT, then this could potentially result in a major development in the treatment of negative symptoms of schizophrenia. Qualified Dance Movement Psychotherapists would require only minimal additional training before they would be able to administer the treatment, and there are also qualified Body Psychotherapists who are able to deliver BPT in groups. Given that the intervention is manualised it can, in theory, be delivered in a consistent and reliable manner. Appropriate spaces as well as the required equipment are likely to be available in most services. Thus, it should be possible to roll out the intervention in the NHS and in mental health services in other countries, and widely offer group BPT to large numbers of patients at limited costs.

In case the trial shows that BPT is effective, this will also be a reason to explore in more depth the potential therapeutic mechanisms involved in the treatment process, such as the linked experience of movement and emotion and the socio-therapeutic facilitation of group interactions through the body oriented interventions. It will also be important to see whether and, if so, to what extent lower negative symptoms translate into improved social contacts and a better quality of life. The latter issue will be addressed in the analysis of secondary outcomes.

## Abbreviations

BPT: Body Psychotherapy; PANSS: Positive and Negative Symptom Scale; NESS: Negative symptoms of schizophrenia BPT trial; NICE: National Institute for Clinical Excellence; DMEC: Data Monitoring and Ethics Committee; TSC: Trial Steering Committee; REC: Research Ethics Committee; CAINS: Clinical Assessment Interview for Negative Symptoms; CSRI: Client Service Receipt Inventory; QALY: Quality Adjusted Life Years; NHS: National Health Service; CEAC: Cost-effectiveness acceptability curve; TAU: Treatment As Usual.

## Competing interests

FR owns the copyright to the BPT manual which is used in the trial.

## Authors’ contributions

All authors have read and approved the final manuscript. SP coordinated and produced the initial outline of the application, is chief investigator for the ‘NESS’ study, and was involved in the development of the protocol. UR prepared the initial draft of the proposal and contributed to the design and development of the protocol. TW, CL and RB contributed to the initial design and development of the protocol. MS contributed to the development of the protocol and its preparation for publication. SE contributed to the methodology and analysis plan. PM prepared the plans for the economic analysis.

## Pre-publication history

The pre-publication history for this paper can be accessed here:

http://www.biomedcentral.com/1471-244X/13/26/prepub
